# Endocrine FGFs: Evolution, Physiology, Pathophysiology, and Pharmacotherapy

**DOI:** 10.3389/fendo.2015.00154

**Published:** 2015-09-29

**Authors:** Nobuyuki Itoh, Hiroya Ohta, Morichika Konishi

**Affiliations:** ^1^Medical Innovation Center, Kyoto University Graduate School of Medicine, Kyoto, Japan; ^2^Department of Microbial Chemistry, Kobe Pharmaceutical University, Kobe, Japan

**Keywords:** biomarker, disease, endocrine, FGF, Klotho, metabolism, mutation, polymorphism

## Abstract

The human fibroblast growth factor (FGF) family comprises 22 structurally related polypeptides that play crucial roles in neuronal functions, development, and metabolism. FGFs are classified as intracrine, paracrine, and endocrine FGFs based on their action mechanisms. Paracrine and endocrine FGFs are secreted signaling molecules by acting via cell-surface FGF receptors (FGFRs). Paracrine FGFs require heparan sulfate as a cofactor for FGFRs. In contrast, endocrine FGFs, comprising FGF19, FGF21, and FGF23, require α-Klotho or β-Klotho as a cofactor for FGFRs. Endocrine FGFs, which are specific to vertebrates, lost heparan sulfate-binding affinity and acquired a systemic signaling system with α-Klotho or β-Klotho during early vertebrate evolution. The phenotypes of endocrine *FGF* knockout mice indicate that they play roles in metabolism including bile acid, energy, and phosphate/active vitamin D metabolism. Accumulated evidence for the involvement of endocrine FGFs in human genetic and metabolic diseases also indicates their pathophysiological roles in metabolic diseases, potential risk factors for metabolic diseases, and useful biomarkers for metabolic diseases. The therapeutic utility of endocrine FGFs is currently being developed. These findings provide new insights into the physiological and pathophysiological roles of endocrine FGFs and potential diagnostic and therapeutic strategies for metabolic diseases.

## Introduction

**Figure 1:**
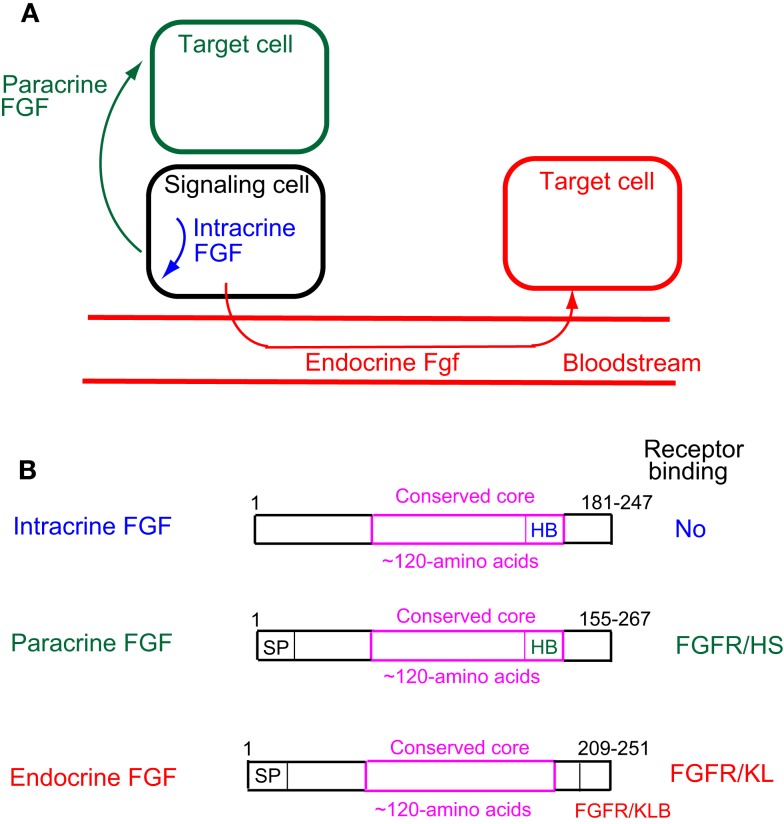
**(A)** Action mechanisms of FGFs. FGFs act on target cells in an intracrine, paracrine, or endocrine manner. **(B)** Schematic representations of FGF structures. SP, HB, and FGFR/KB indicate a secreted signal sequence, heparan sulfate-binding site, and FGFR/Klotho complex-binding site, respectively.

Cell–cell communication with secreted signaling molecules ensures proper development and metabolism. Most fibroblast growth factors (FGFs) are well-established secreted signaling proteins that play critical roles in development and metabolism. The FGF family comprises 22 structurally and evolutionarily related proteins including FGF1–FGF23 in humans and mice (1). The human and mouse FGF families do not include FGF15 and FGF19, respectively, as they are orthologs. FGFs are classified as intracrine, paracrine, and endocrine FGFs by their action mechanisms (Figure 1A). Paracrine and endocrine FGFs, which comprise 15 and 3 FGFs, respectively, are secreted signaling proteins. In contrast, intracrine FGFs, which comprise four FGFs, are intracellular proteins that are not functionally related to paracrine or endocrine FGFs (1).

Endocrine FGFs comprise FGF19, FGF21, and FGF23. The mouse is a mammalian model that is useful for investigating gene functions through gene targeting. All endocrine *FGF* knockout mice have been generated and extensively analyzed, with their phenotypes indicating important roles in metabolism (1). Evidence to support the involvement of endocrine FGFs in human inherited and metabolic diseases has also been accumulated. We herein have reviewed the physiological/pathophysiological roles and diagnostic/therapeutic utility of endocrine FGFs.

## Identification of Endocrine FGFS

*FGF15* was originally identified as a downstream target of the chimeric homeodomain oncoprotein E2A-Pbx1 in mice (2). *FGF19* was also originally identified by a homology-based DNA database search in humans (3, 4). However, *FGF19* was later found to be the human ortholog of mouse *FGF15* (5). Only rodent orthologs were named *FGF15*. Other vertebrate orthologs were named *FGF19*.

*FGF21* was originally identified by a homology-based polymerase chain reaction in mice and humans (6). FGF21 was later found to be a stimulator of glucose uptake in cultured mouse adipocytes (7). *FGF21* was also identified as a hepatic gene inducible by fasting or a ketogenic diet in mice (8, 9).

*FGF23* was originally identified by a homology-based DNA database search in mice and humans (10). Human *FGF23* was simultaneously identified as a gene responsible for autosomal dominant hypophosphatemic rickets (ADHR) characterized by low serum phosphate levels, rickets, osteomalacia, lower extremity deformities, a short stature, bone pain, and dental abscesses (11). Human *FGF23* was subsequently found to be a causative humoral protein for human tumor-induced osteomalacia characterized by hypophosphatemia caused by renal phosphate wasting (12).

## Unique Actions of Endocrine FGFS with FGF Receptors and Klotho Coreceptors

Paracrine and endocrine FGFs, which are extracellular signaling molecules, bind to and activate cell-surface FGF receptors (FGFRs). The human *FGFR* gene family comprises four members, *FGFR1*–*FGFR4*, that encode single-pass transmembrane proteins of ~800 amino acids with three extracellular immunoglobulin-like domains I, II, and III and a split intracellular tyrosine kinase domain. *FGFR1*, *FGFR2*, and *FGFR3* genes encode two alternative splicing variants (b and c) of immunoglobulin-like domain III, which is a determinant of ligand-binding specificities (1). Thus, there are seven FGFR proteins, FGFRs 1b, 1c, 2b, 2c, 3b, 3c, and 4, with distinct ligand-binding specificity. The functional dimerization and transphosphorylation of FGFRs induced by FGF binding result in the activation of FGFRs, followed by the activation of intracellular FGF signaling pathways including RAS–RAF–MAPK, PI3K–AKT, STAT, and PLCγ pathways (1).

Paracrine FGFs bind to heparan sulfate proteoglycans, which extracellularly localize at neighboring cells as cofactors for FGFRs. Binding to heparan sulfate proteoglycans is essential for FGF signaling in a paracrine manner (Figure 1B) (1). In contrast, endocrine FGFs with reduced heparan sulfate-binding affinity are unable to function in a paracrine manner. Endocrine FGFs require α-Klotho or β-Klotho as a cofactor for FGFRs to function in an endocrine manner (1). α-Klotho and β-Klotho, which share structural similarities and characteristics with each other, are single-pass transmembrane proteins of ~1,000 amino acids with a short cytoplasmic domain (13). However, endocrine FGFs cannot efficiently bind to FGFR, α-Klotho, or β-Klotho alone; they efficiently bind to the FGFR–Klotho complex. The binding site for the FGFR–Klotho complex is localized in the C-terminal tail of endocrine FGF (Figure 1B) (14). FGF19 activates FGFR4 with β-Klotho and FGF21 activates FGFR1c with β-Klotho, whereas FGF23 activates FGFR1c with α-Klotho (1). FGF19 and FGF23 exhibit metabolic and proliferative activities. However, FGF21 is a unique FGF with metabolic activity, but no proliferative activity (15).

## The Evolutionary History of Vertebrate-Specific Endocrine *FGF* Genes

**Figure 2:**
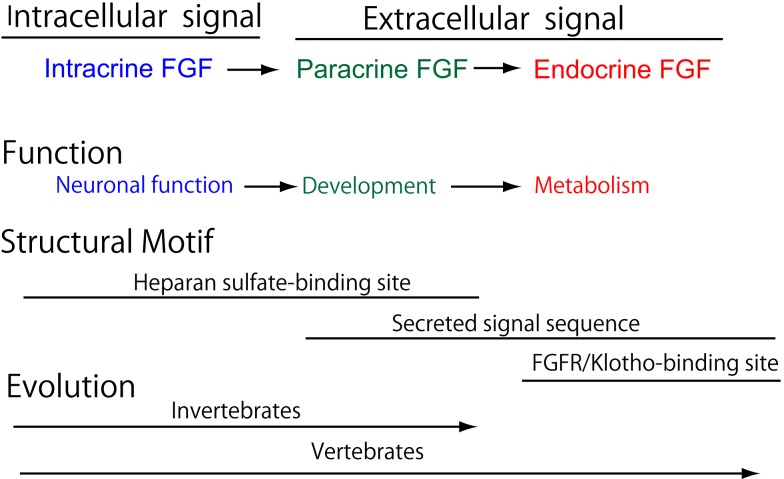
**The functional evolutionary history of the FGF family**.

The FGF signaling system has been conserved throughout metazoan evolution. The evolutionary history of the *FGF* gene family has already been proposed (5). The founder of the *FGF* family is an intracrine *FGF*. Intracrine FGFs, which are not secreted proteins, possess heparan sulfate-binding sites, but no secreted signal sequences. The prototype of the paracrine *FGF* subfamily was generated from the founder by gene duplication events early in metazoan evolution. During this evolution, paracrine FGFs acquired secreted signal sequences, thereby allowing them to function as extracellular FGFs in a paracrine manner. Intracrine and paracrine *FGF*s have been identified in invertebrates and vertebrates. In contrast, endocrine *FGF*s have been detected in vertebrates, but not invertebrates, indicating that they are specific to vertebrates (Figure 2). The prototypic endocrine *FGF* was generated from the prototype of paracrine *FGF* by local gene duplication early in vertebrate evolution. *FGF19*, *FGF21*, and *FGF23* were subsequently generated from the prototype by two large-scale genome duplication events early in vertebrate evolution (5). Endocrine FGFs lost heparan sulfate-binding affinity and acquired FGFR/Klotho complex-binding affinity for a systemic signaling system in an endocrine manner during their evolution (5). *FGF19* and *FGF23* have been identified in all vertebrates examined, including teleosts, amphibians, reptiles, birds, and mammals. However, *FGF21* was not detected in any birds examined, indicating that *FGF21* was lost in the avian lineage (16, 17). The avian genome is principally characterized by its constrained size with the loss of many genes in the avian lineage, indicating that pan-avian genomic diversity covaries with adaptations to different lifestyles and the convergent evolution of traits (18).

## Roles of Endocrine FGFs Indicated by Their Knockout Mouse Phenotypes

The mouse is a mammalian model that is useful for investigating gene functions. All endocrine *FGF* knockout mice have been extensively analyzed, indicating their important physiological roles in metabolism (Table 1).

**Table 1:** **Roles of endocrine FGFs indicated by their knockout mouse phenotypes**.

FGF15	Morphogenesis of the cardiac outflow tract (2, 19), hepatic bile acid metabolism (20), gallbladder filling (21), hepatic protein and glycogen metabolism (22), liver regeneration (23, 24), and fibrosis-induced hepatocellular carcinogenesis (25)
FGF21	Lipolysis in white adipose tissue (28), adaptation to a ketogenic diet (29, 30), upstream effector of adiponectin (31, 32), glucagon action (33), pancreatic beta-cell proliferation and insulin synthesis (34), adaptation to ER stress (35), cardiac hypertrophy (36, 37), pancreatitis (38), diabetic cardiomyopathy (39), and aortic remodeling (40)
FGF23	Renal phosphate and active vitamin D metabolism (41) and ear development and function (42)

### FGF15

*FGF15* knockout mice survive until E10.5, but then gradually die due to defects in the cardiac outflow tract, indicating that FGF15 is required for proper morphogenesis of the cardiac outflow tract during embryonic development in the heart (2, 19). *FGF15* is selectively expressed in the ileum at postnatal stages, and a small number of *FGF15* knockout mice survive until postnatal stages, which suggests that FGF15 plays crucial roles in regulating hepatic bile acid synthesis and gallbladder filling in an endocrine manner (20, 21). Surviving *FGF15* knockout mice also fail to properly maintain blood concentrations of glucose and normal postprandial amounts of liver glycogen, indicating that FGF15 is a postprandial, insulin-independent activator of hepatic protein and glycogen synthesis (22). Surviving hepatectomized *FGF15* knockout mice exhibit severe defects in liver regeneration, indicating that FGF15 also plays a role in liver regeneration (23, 24). Ileal *FGF15* expression is increased in mice undergoing carcinogenesis. Fibrosis-induced hepatocellular carcinogenesis results in fewer and smaller tumors in surviving *FGF15* knockout mice than in wild-type mice, suggesting that ileal FGF15 enhances the development of carcinoma (25).

### FGF21

*FGF21* is expressed in the liver, pancreas, white adipose tissue, and muscle of mice (6, 26, 27). The phenotypes of *FGF21* knockout mice, which are viable and fertile, indicate that FGF21 stimulates lipolysis in the white adipose tissue during feeding, but inhibits it during fasting (28). *FGF21* knockout mice fed a ketogenic diet also indicate that FGF21 plays a role in the adaptation to a ketogenic diet and impairs adipocyte insulin sensitivity (29, 30). *FGF21* knockout mice also indicate that FGF21 is an upstream effector of adiponectin in white adipocytes and that adiponectin also mediates many of the systemic effects of FGF21 on energy metabolism and insulin sensitivity in the liver and skeletal muscle (31, 32). Glucagon, an essential regulator of glucose homeostasis, modulates lipid metabolism and promotes weight loss. Glucagon receptor activation increases hepatic *FGF21* expression in mice. *FGF21* knockout mice indicate that FGF21 contributes, at least in part, to glucose, energy, and lipid metabolism controlled by glucagon (33). *FGF21* knockout mice also exhibit insulin resistance, while being normoglycemic, and this is associated with increases in pancreatic beta-cell proliferation and insulin synthesis, which act as compensatory responses. This resistance results from enhanced growth hormone sensitivity in *FGF21* knockout pancreatic islets, indicating that FGF21 is important in the regulation of pancreatic beta-cell proliferation and insulin synthesis, possibly via the modulation of growth hormone signaling (34). Endoplasmic reticulum (ER) stress leads to the development and progression of various diseases such as obesity and diabetes. ER stress induces hepatic *FGF21* expression. ER stress and hepatic lipid accumulation are increased in *FGF21* knockout mice, indicating that FGF21 plays a role in the adaptive responses to ER stress (35). *FGF21* knockout mice also have an increased relative heart weight and develop enhanced signs of dilatation. FGF21 is markedly secreted by cardiac cells in response to cardiac stress. Thus, the heart appears to be a target of locally generated FGF21, indicating that FGF21 acts on cardiomyocytes, possibly in a paracrine manner (36). FGF21 prevents the production of reactive oxygen species in cardiac cells by acting as an antioxidant factor in the heart, thereby preventing pro-oxidative pathways under inflammatory or hypertrophic conditions (37). The expression of *FGF21* in pancreatic acinar cells is markedly increased during cerulein-induced pancreatitis. *FGF21* knockout mice have higher serum amylase levels and more tissue damage, suggesting a novel function for FGF21 as an immediate response gene protecting pancreatic acini from overt damage (38). *FGF21* knockout mice with streptozotocin-induced diabetes exhibit severe cardiac dysfunction, increased cardiac lipid accumulation, and severe aortic remodeling, indicating that FGF21 plays roles in the development and progression of diabetic cardiomyopathy and aortic remodeling (39, 40).

### FGF23

*FGF23* is mainly expressed in osteocytes in mice. *FGF23* knockout mice survive until birth, but then gradually die by 11 weeks after birth. The phenotypes of *FGF23* knockout mice indicate that osteocytic FGF23 is a regulator for phosphate and active vitamin D metabolism in the kidney (41). FGF23 is also ubiquitously expressed throughout the cochlea. *FGF23* heterozygous knockout mice are profoundly deaf with moderate hearing loss and a near-normal morphology. However, *FGF23* homozygous knockout mice have dysplastic bulla and ossicles. These findings indicate that FGF23 is critical for normal development of the middle ear as well as the functions of the middle and inner ear (42).

## Roles of Endocrine FGFs Indicated by Human Diseases

Evidence has been accumulated to support the involvement of FGF19 and FGF23 in human diseases and also indicates their crucial pathophysiological roles in diseases (Table 2).

**Table 2:** **Roles of endocrine FGFs indicated by human diseases**.

**Inherited disease with loss-of-function mutations**	
FGF23	Hyperphosphatemic familial tumoral calcinosis (45, 46)
**Inherited disease with gain-of-function mutations**	
FGF23	Autosomal dominant hypophosphatemic rickets (11, 47)
**Paraneoplastic diseases with gain-of-function**	
FGF19	Extrahepatic cholestasis (43)
FGF23	Osteomalacia (12)
**SNPS as potential risk factors for diseases**	
FGF21	Intake of dietary macronutrients (48), metabolic syndrome, obesity, and diabetes (49)
FGF23	Cardiac abnormalities in Kawasaki disease (50) and prostate cancer (51)
**Serum levels as biomarkers for diseases**	
FGF19	Renal failure (52, 53), coronary artery disease (54), intestinal failure-associated liver disease (55), diabetes (56), Crohn’s disease (57, 58), and prostate cancer (60)
FGF21	Renal failure (61), cardiovascular disease (62–64), mitochondrial disease (65, 66), obesity and diabetes (56, 67–70), anorexia nervosa (71), Cushing’s syndrome (72), Brown adipose tissue activity (73), non-alcoholic fatty liver disease (74, 75), chronic hepatitis C with steatosis (76), systemic inflammatory response syndrome (77), and rheumatoid arthritis (78)
FGF23	Renal failure (79–81), cardiovascular failure (82–86), and stroke (87, 88)

### FGF19

#### Paraneoplastic Diseases with Gain-of-Function

Extrahepatic cholestasis is a condition in which the release of bile acid is blocked in the bile ducts. Cholestasis is caused by a pancreatic tumor. Markedly increased serum FGF19 levels and the abundant expression of hepatic *FGF19* are observed in cholestatic patients, indicating that FGF19 signaling is involved in some of the adaptations that protect the liver against bile acid toxicity (43).

### FGF23

#### Inherited Disease with Loss-of-Function Mutations

Hyperphosphatemic familial tumoral calcinosis (HFTC) is a rare autosomal recessive metabolic disorder characterized by the deposition of calcium phosphate crystals. HFTC was initially attributed to loss-of function mutations in *GLANT3* encoding a glycosyltransferase (44). Loss-of-function mutations in *FGF23* are also associated with HFTC (45, 46).

#### Inherited Disease with Gain-of-Function Mutations

Autosomal dominant hypophosphatemic rickets is characterized by isolated renal phosphate wasting, hypophosphatemia, and inappropriately normal 1,25-dihydroxyvitamin D3 levels. Gain-of-function mutations in *FGF23* result in AHDR. Cleaved FGF23 forms due to intracellular proteolysis lose their biological activities. *FGF23* mutations in ADHR result in impaired proteolysis of FGF23, resulting in increased serum levels of active FGF23 (11, 47).

#### Paraneoplastic Diseases with Gain-of-Function

Osteomalacia is characterized by the softening of bones due to defective bone mineralization. Tumors that overexpress *FGF2*3 induce tumor-induced osteomalacia characterized by hypophosphatemia, which is caused by renal phosphate wasting (12).

## Single-Nucleotide Polymorphisms in Endocrine *FGF* Genes as Potential Risk Factors for Diseases

Single-nucleotide polymorphisms (SNPs) are DNA sequence variations that commonly occur within a population (e.g., 1%) with a single nucleotide in the genome. These SNP variations underlie differences in the susceptibility of humans to diseases. The severity of illnesses and the responses of the human body to treatments are also manifestations of SNPs. SNPs in endocrine *FGF*s also suggest their potential roles in risk factors for diseases (Table 2).

### FGF21

The intake of dietary macronutrients such as carbohydrates, protein, and fat is associated with the risk of obesity and diabetes. A SNP located in the exon of *FGF21* is significantly associated with the percentage of total caloric intake from protein and carbohydrates, indicating that *FGF21* is a potential susceptibility gene for obesity and diabetes (48). SNPs in the 3′ untranslated region of *FGF21* are also associated with metabolic syndrome, obesity, and diabetes (49). However, the mechanisms underlying these associations currently remain unclear.

### FGF23

Kawasaki disease, which is a systemic panvasculitis that affects all medium/small-sized vessels, mainly occurs in early childhood and increases the risk of cardiac abnormalities. A SNP in the intron of *FGF23* is significantly associated with higher serum FGF23 levels and cardiac abnormalities in children with Kawasaki disease (50). Three distinct SNPs in *FGF23* are also associated with an increased risk of prostate cancer, indicating that *FGF23* genetic variations increase prostate cancer susceptibility (51).

## Serum Endocrine FGF Levels as Biomarkers for Diseases

Endocrine FGFs mainly play roles in an endocrine manner via the blood circulation system. Serum endocrine FGFs levels are sometimes altered in patients with metabolic and other diseases, indicating that endocrine FGFs are biomarkers for these diseases (Table 2).

### FGF19

#### Renal Failure

Renal failure is a condition in which the kidneys fail to adequately filter waste products from the blood. Serum FGF19 levels are significantly increased in end-stage renal failure patients with chronic hemodialysis (52). The postprandial FGF19 response in serum is blunted in chronic kidney disease and is also associated with impaired insulin and C-peptide signaling (53).

#### Cardiovascular Disease

Coronary artery disease (CAD) is a group of diseases including stable angina, unstable angina, myocardial infarction, and sudden coronary death. Serum FGF19 levels are decreased in CAD in relation to its severity, suggesting that reductions in the expression of *FGF19* are involved in the development and progression of CAD (54).

#### Intestinal Failure-Associated Liver Disease

In pediatric intestinal failure (IF), serum FGF19 levels are decreased corresponding to IF-associated liver disease (IFALD). This suggests that FGF19 contributes to the pathogenesis of IFALD (55).

#### Energy Metabolism Disorder

Serum FGF19 levels are decreased in patients with type 2 diabetes, indicating that the FGF19 pathway is significantly perturbed in severe diabetes (56).

#### Bile Acid Metabolism Disorder

Bile acids are reabsorbed by the terminal ileum. However, their reabsorption is frequently impaired in Crohn’s disease, resulting in secretory diarrhea. Serum FGF19 levels are decreased in Crohn’s disease associated with diarrhea and disease activity, indicating that FGF19 has potential as a biomarker for the functioning ileum in Crohn’s disease (57, 58). However, serum FGF19 levels are not affected in gallstone carriers (59).

#### Cancer

Serum FGF19 levels in the high Gleason grade of prostate cancer are higher than those in the low Gleason grade group, indicating that FGF19 promotes the progression of prostate cancer (60).

### FGF21

#### Renal Failure

Serum FGF21 levels are increased in chronic kidney disease and acute kidney dysfunction, suggesting that renal excretion is a major route for the elimination of FGF21 (61).

#### Cardiovascular Disease

Coronary heart disease (CHD) is characterized by the narrowing of small blood vessels in the heart, which may lead to heart attacks. Serum FGF21 levels are significantly increased in CHD. Serum FGF21 levels in CHD patients with diabetes, hypertension, or both are higher than those in patients without these comorbidities. High serum FGF21 levels are associated with adverse lipid profiles in CHD patients, indicating that the paradoxical increase in serum FGF21 levels in CHD patients is a compensatory response or resistance to FGF21 (62). Serum FGF21 levels are positively correlated with carotid intima-media thickness (IMT) in women, but not in men, indicating that elevated serum FGF21 levels in women are an independent risk factor for increased carotid IMT (63). Preeclampsia, a serious cardiovascular complication in pregnancy, is associated with an increased future metabolic and cardiovascular risk. Serum FGF21 levels are significantly increased in patients with preeclampsia during pregnancy (64).

#### Mitochondrial Disease

Mitochondrial disease caused by dysfunctional mitochondria is a group of disorders with highly variable phenotypes including mitochondrial myopathy, diabetes mellitus, deafness, optic neuropathy, and multiple sclerosis-type disease. Serum FGF21 levels are significantly increased in human mitochondrial disease and the best predictor of mitochondrial disease among classical indicators including creatine kinase, lactate, and ­pyruvate (65, 66).

#### Energy Metabolism Disorder

Serum FGF21 levels are significantly increased in patients with obese and type 2 diabetes, suggesting the direct positive metabolic effects of FGF21 (56, 67–70). Anorexia nervosa (AN) is an eating disorder that is characterized by a low weight and food restriction. Serum FGF21 levels are significantly decreased in AN, suggesting that FGF21 is involved in the pathophysiology of or a complex adaptive response to AN (71). Cushing’s syndrome is caused by prolonged exposure to inappropriately high levels of glucocorticoids. Serum FGF21 levels are increased in patients with Cushing’s syndrome. Increased serum FGF21 levels are attributed to their excessive fat accumulation and related metabolic abnormalities, but not to the direct effects of cortisol on the production of FGF21 (72). Brown adipose tissue, which contains a markedly higher number of mitochondria, generates body heat in animals without shivering. Since brown adipose tissue is expected to markedly contribute to energy homeostasis in humans, it may represent a therapeutic target of obesity. Serum FGF21 levels are associated with brown adipose tissue activity, indicating a novel mechanism by which brown adipose tissue activity may be enhanced (73).

#### Lipid Metabolism Disorder

Serum FGF21 levels are increased in patients with non-alcoholic fatty liver disease (NAFLD), which supports the role of FGF21 as a key regulator of hepatic lipid metabolism (74). In contrast to adults with NAFLD, serum FGF19 and FGF21 levels are inversely associated with hepatic damage in children with NAFLD, providing insights for a better understanding of the progression of NAFLD (75).

#### Infection and Inflammation

Hepatitis C (HC) is an infectious disease that is caused by the HC virus. Serum FGF21 levels are increased in chronic HC (CHC) patients with steatosis and are associated with the steatosis grade, which may be a useful diagnostic marker for determining hepatic steatosis in CHC (76). Sepsis is an illness in which the body has a severe response to infection. Systemic inflammatory response syndrome (SIRS) is an inflammatory state in the whole body due to the response of the immune system to infection. SIRS is also closely related to sepsis. Serum FGF21 levels are significantly increased in patients with sepsis and SIRS, suggesting a role for FGF21 in inflammation (77). Rheumatoid arthritis (RA) is a chronic inflammatory disease in small hand and foot joints. Serum FGF21 levels are significantly increased in patients with RA, indicating the compensatory response of FGF21 to inflammation and immune response (78).

### FGF23

#### Renal Failure

Serum FGF23 levels are significantly increased in chronic kidney disease, indicating the important role of FGF23 in maintaining a neutral phosphate balance as the glomerular filtration rate decreases (79–81).

#### Cardiovascular Disease

Serum FGF23 levels are positively correlated with the risk of heart failure (82). Serum FGF23 levels are increased in children with heart failure (83). These findings indicate that increased serum FGF23 levels are a risk factor for heart failure. Serum FGF23 levels are also increased in association with angiographic severity and the extent of CAD (84). Increased serum FGF23 levels are also a risk factor for myocardial infraction and hemorrhagic stroke (85). Serum FGF23 levels are strongly associated with left ventricular hypertrophy (86). These findings provide additional evidence for the role of FGF23 in cardiovascular disease.

#### Stroke

Stroke is a disease that is caused by cutting off the blood supply to part of the brain (ischemic) or by bleeding into the surrounding brain tissue from a weakened vessel (hemorrhagic). Serum FGF23 levels are increased in patients with stroke (87, 88).

## Therapeutic Utility of Endocrine FGFs in Humans or Monkeys

### FGF19

FGF19 is expected to prevent bile acid-induced liver damage by reducing hepatic bile acid levels through the modulation of bile acid synthesis. However, since FGF19 also stimulates the proliferation of hepatocytes, the potential risk associated with prolonged exposure to supraphysiological levels of FGF19 represents a major hurdle in the development of an FGF19-based therapy in hepatocellular carcinogenesis. A manufactured FGF19 variant, M70, which carries three amino acid substitutions and a five-amino acid deletion, regulates bile acid metabolism without tumorigenicity (89). The administration of M70 to healthy human volunteers potently reduces serum levels of 7α-hydroxy-4-cholesten-3-one, a surrogate marker for the hepatic activity of cholesterol 7α-hydroxylase (CYP7A1), which catalyzes the rate-limiting step in the bile acid synthetic pathway. These findings indicate that the development of non-tumorigenic FGF19 variants represents an effective approach for the prevention and treatment of cholestatic liver diseases and other disorders associated with bile acid dysregulation (89) (Table 3).

**Table 3:** **Therapeutic utility of endocrine FGFs in humans or monkeys**.

FGF19	Cholestatic liver disease and bile acid dysregulation (89)
FGF21	Diabetes and metabolic disease (90)
FGF21 variant (LY2405319)	Obesity and metabolic disease (91, 92)
FGF21 variant (Fc-FGF21)	Diabetes (93)
FGF21 variant (PF-05231023)	Diabetes (94)
FGF21 Avimer	Obesity (95)
Anti-FGF23 (KRN23)	X-linked hypophosphatemia (96, 97)

### FGF21

The daily administration of FGF21 to diabetic rhesus monkeys for 6 weeks induces marked reductions in fasting serum glucose, triglyceride, insulin, and glucagon levels, significant improvements in lipoprotein profiles, and significant weight loss, thereby supporting the development of FGF21 for the treatment of diabetes and other metabolic diseases in humans (90). LY2405319 (LY) is a manufactured FGF21 variant with significantly improved biopharmaceutical properties (91). The administration of LY to patients with obesity and type 2 diabetes for 28 days produces significant improvements in dyslipidemia, a shift to a potentially less atherogenic apolipoprotein concentration profile, and favorable effects on body weight. These findings indicate that FGF21-based therapies may be effective for the treatment of selected metabolic disorders (92).

FGF21 has a short half-life (1–2 h). Fc-FGF21 is a long-acting FGF21 variant that is generated by fusing with an Fc fragment. In obese rhesus monkeys treated with FGF21 (once a day) or Fc-FGF21 (once a week), serum glucose, insulin, cholesterol, and triglyceride levels and body weight are markedly lower with Fc-FGF21 than with FGF21, indicating that the administration of Fc-FGF21 once a week leads to similar or greater efficacy than FGF21 administered daily (93). PF-05231023 is also a long-acting FGF21 variant generated by covalently conjugating FGF21 molecules to a non-targeting human IgG1 κ scaffold. Single intravenous injection of PF-05231023 to type 2 diabetes mellitus patients decreases serum triglyceride, total cholesterol, and low-density lipoprotein cholesterol levels and increases high-density lipoprotein cholesterol levels, but have no apparent effect on glucose levels (94).

As an alternative to FGF21, bispecific Avimer polypeptides that bind with high affinity and specificity to one of the receptor and coreceptor pairs used by FGF21, FGFR1c, and β-Klotho have been generated. These Avimers exhibit FGF21-like activity with greater potency than FGF21 *in vitro*. In obese male cynomolgus monkeys, a bispecific Avimer exhibits improved metabolic parameters and leads to greater reductions in body weight than those with FGF21, thereby supporting a novel therapeutic approach to target this potentially important pathway in the treatment of diabetes and obesity (95).

### FGF23

X-linked hypophosphatemia (XLH) with elevated serum FGF23 levels is the most common heritable form of rickets and osteomalacia. KRN23 is a human anti-FGF23 antibody that has been developed as a potential treatment for XLH patients. KRN23 significantly increases the maximum renal tubular threshold for phosphate reabsorption, serum phosphate, and 1,25-dihydroxyvitamin D in XLH patients, suggesting utility for KRN23 in XLH patients (96, 97).

## Conclusion

Endocrine FGFs comprising FGF19, FGF21, and FGF23 require FGFR and α-Klotho or β-Klotho as a cofactor for FGFR for their signaling in an endocrine manner. Endocrine FGFs lost heparan sulfate-binding affinity and acquired systemic signaling system with α-Klotho or β-Klotho during their evolution. The phenotypes of endocrine *FGF* knockout mice indicate that endocrine FGFs play important physiological roles in metabolism including bile acid metabolism, energy metabolism, and phosphate/active vitamin D metabolism. Evidence for the involvement of endocrine FGFs in human inherited and metabolic diseases has also been increasing, indicating their crucial pathophysiological roles in metabolic diseases, their SNPs as potential risk factors for diseases, and their serum levels as useful biomarkers for metabolic diseases. Furthermore, the therapeutic utility of endocrine FGFs is currently being developed. These findings provide new insights into the physiological and pathophysiological roles of endocrine FGFs and may provide potential diagnostic and therapeutic strategies for metabolic diseases.

## Conflict of Interest Statement

The authors declare that the research was conducted in the absence of any commercial or financial relationships that could be construed as a potential conflict of interest.
